# Cold Exposure Can Induce an Exaggerated Early-Morning Blood Pressure Surge in Young Prehypertensives

**DOI:** 10.1371/journal.pone.0150136

**Published:** 2016-02-26

**Authors:** Cian-Hui Hong, Terry B. J. Kuo, Bo-Chi Huang, Yu-Cheng Lin, Kuan-Liang Kuo, Chang-Ming Chern, Cheryl C. H. Yang

**Affiliations:** 1 Institute of Brain Science, National Yang-Ming University, Taipei, Taiwan; 2 Sleep Research Center, National Yang-Ming University, Taipei, Taiwan; 3 Institute of Biophotonics, National Yang-Ming University, Taipei, Taiwan; 4 Department of Neurology, Taipei Veterans General Hospital, Taipei, Taiwan; 5 Department of Family Medicine, Taipei City Hospital, Taipei, Taiwan; 6 Brain Research Center, National Yang-Ming University, Taipei, Taiwan; 7 Department of Education and Research, Taipei City Hospital, Taipei, Taiwan; 8 Institute of Translational and Interdisciplinary Medicine, National Central University, Taoyuan, Taiwan; University of Bologna, ITALY

## Abstract

Prehypertension is related to a higher risk of cardiovascular events than normotension. Our previous study reported that cold exposure elevates the amplitude of the morning blood pressure surge (MBPS) and is associated with a sympathetic increase during the final sleep transition, which might be critical for sleep-related cardiovascular events in normotensives. However, few studies have explored the effects of cold exposure on autonomic function during sleep transitions and changes of autonomic function among prehypertensives. Therefore, we conducted an experiment for testing the effects of cold exposure on changes of autonomic function during sleep and the MBPS among young prehypertensives are more exaggerate than among young normotensives. The study groups consisted of 12 normotensive and 12 prehypertensive male adults with mean ages of 23.67 ± 0.70 and 25.25 ± 0.76 years, respectively. The subjects underwent cold (16°C) and warm (23°C) conditions randomly. The room temperature was maintained at either 23°C or 16°C by central air conditioning and recorded by a heat-sensitive sensor placed on the forehead and extended into the air. BP was measured every 30 minutes by using an autonomic BP monitor. Electroencephalograms, electrooculograms, electromyograms, electrocardiograms, and near body temperature were recorded by miniature polysomnography. Under cold exposure, a significantly higher amplitude of MBPS than under the warm condition among normotensives; however, this change was more exaggerated in prehypertensives. Furthermore, there was a significant decrease in parasympathetic-related RR and HF during the final sleep transition and a higher early-morning surge in BP and in LF% among prehypertensives, but no such change was found in normotensives. Our study supports that cold exposure might increase the risk of sleep-related cardiovascular events in prehypertensives.

## Introduction

An exaggerated morning blood pressure surge (MBPS) change is probably a risk factor for the development of a cardiovascular event [1–3]. A previous study demonstrated a link between the occurrence of cardiovascular events and a low ambient temperature in addition to the MBPS [4]. Cardiovascular events occur more frequently on winter mornings [5, 6], and a low ambient temperature has been regarded as a major contributor [7]. The peak incidence of many cardiovascular events is at the end of the sleep period and prior to morning awakening, rather than at any other period of the day [8–10]. Blood pressure (BP) variation is strictly regulated within a narrow range by the autonomic nervous system (ANS). Decreased parasympathetic activity together with increased sympathetic activity is presumed to cause cardiovascular abnormalities [1, 11, 12].

According to the *Seventh Report of the Joint National Commission* (*JNC*-7), prehypertension (120 to 139 mmHg systolic/80 to 89 mmHg diastolic) has been established as a new BP category. The prehypertensive population is a high normal BP population and has been indicated to have a high risk of progressing to hypertension [13]. Furthermore, the results of clinical trials have suggested that prehypertension is associated with an increased risk of cardiovascular events [14, 15], hypertension, a higher body mass index, and diabetes mellitus [16, 17]. Our recent study of normotensives reported the association of cold exposure with an acute increase in sympathetic activity during the sleep stage transition from non-rapid eye movement (NREM) to rapid eye movement (REM) sleep that occurs late in sleep; this, in turn, is related to an exaggerated MBPS after morning awakening [18]. Therefore, the interaction of cold exposure, sleep, and ANS functioning is likely to be involved in sleep-related cardiovascular events.

The application of heart rate variability (HRV) analysis to freely moving humans and animals has recently gained attention as a means of quantifying ANS functioning noninvasively [19–22]. Spectral analysis of HRV by Fourier transformation has been categorized into high-frequency (HF) and low-frequency (LF) powers. HF is considered to represent vagal control of the heart rate [19, 23]. The LF% and LF/HF ratio have been considered by some investigators to reflect sympathetic modulations or alternatively to mirror the sympathovagal balance [19–22].

Thus far, the effects of cold exposure in prehypertensives on changes in ANS functioning during sleep stage transitions and during the MBPS have not been investigated. Prehypertension is prevalent among younger individuals and is associated with hypertension during later life [24, 25]. Thus, the present study evaluated the effects of cold exposure on autonomic functioning and the MBPS among young prehypertensives.

## Materials and Methods

### Subjects

All the procedures used in this study were approved by the Institutional Review Board of National Yang-Ming University. Based on the classification of BP in the *JNC-7*, the subjects were classified into two groups: normotensives and prehypertensives. Twelve normotensive male adults and 12 prehypertensive male adults were recruited from a university population; they had mean ages of 23.67 ± 0.70 and 25.25 ± 0.76 years, respectively. The demographic data of the two groups of subjects are presented in Table 1. Subjects receiving any medication, with any history of psychopathology, with any medical condition that influences sleep or ANS functioning, or with a history of smoking or alcoholism, were excluded from this study. All subjects provided written informed consent to the experimental procedures, which were fully described to them.

**Table 1:** General measurements during baseline recording under the two ambient temperature conditions.

	Normotensives (n = 12)		Prehypertensives (n = 12)	
	Warm	Cold	Warm	Cold
Age (years old)	23.67 ± 0.70		25.25 ± 0.76	
BMI (kg m^-2^)	23.26 ± 0.94	23.26 ± 0.94	23.56 ± 0.53	23.43 ± 0.51
Awake SBP (mmHg)	114.69 ± 1.83	119.14 ± 2.27	127.86 ± 3.60^†^	130.86 ± 2.84^†^
Awake DBP (mmHg)	70.53 ± 1.86	75.06 ± 2.20*	81.06 ± 2.63^†^	84.07 ± 2.83^†^
Sleep SBP (mmHg)	106.84 ± 1.89	106.61 ± 1.95	110.58 ± 2.11	115.11 ± 2.81^†^
Sleep DBP (mmHg)	61.11 ± 0.92	61.28 ± 1.25	64.37 ± 1.89	68.05 ± 2.44*^†^

The values are means ± SEM, *n* = 12, were estimated by measuring the average values from 9:30 pm to 12:00 am. DBP during sleep from prehypertensives under cold exposure were significantly greater than under the warm condition.

Abbreviation: BMI, body mass index; SBP, systolic blood pressure; DBP, diastolic blood pressure.

*p < 0.05 for comparison of the warm condition within the same group

^†^p < 0.05 for comparison with normotensives.

### Data acquisition

Electrophysiological signals were recorded by a miniature physiological signal recorder (TD1, Taiwan Telemedicine Device Company, Taiwan) [23, 26, 27], carried by each subject. The small size (5.2 × 3.1 × 1.2 cm) and light weight (11 g) of the recorder resulted in a minimum level of interference with the lifestyle of the participants. The recording of the electrophysiological signals was a simplified version of standard sleep monitoring [28], with only four electrophysiological signals (electroencephalogram [EEG], electrooculogram [EOG], electromyogram [EMG], and electrocardiogram [ECG]), two temperature signals, and two physical activities signals (ACT) being recorded. Even with only four channels for electrophysiological analysis and sleep scoring, we could nevertheless confirm that a large dataset of results could be gathered, analyzed, and published using only these channels [26]. EEGs were recorded from the C3 point with a reference point at A2 [29]. EOGs were recorded from a pair of differential electrodes placed 1 cm above the right outer canthus and 1 cm below the left outer canthus [28]. The EOG recordings could detect both horizontal and vertical movements of the eyeball in a single recording channel; this approach is widely used in sleep research. EMGs were recorded from a pair of differential electrodes in the submental area. ECGs were recorded from the V5 site on the chest. The room temperature was maintained at either 23°C or 16°C by central air conditioning. The heat-sensitive sensor was placed on the forehead and extended into the air to record the room temperature. A previous study revealed that the personal-level environmental temperature, which is called near body temperature (NBT), is an accurate predictor of the measured BP [30]. Thus, a second sensor, which is surrounded by styrofoam for heat insulation, is often used to assess heat transition resistance ability. This sensor was placed on the chest 4.0 mm from the body's skin and was used to record the temperature between the human body and the bedcover. In this context, we presumed that the changes in the NBT (data not shown) are more crucial in relation to BP measurements and autonomic functioning than the room temperature. All of the acquired information was stored on a flash drive for subsequent off-line analysis. The EEG, EOG, and EMG signals were used for sleep scoring, and the ECG signal was used for HRV analysis.

### Autonomic BP acquisition and uploading system

The cuff of a BP recorder (WatchBP^™^ Home Blood Pressure Monitor, Microlife, Taiwan) was placed on the left upper arm and programmed to collect data at 30-minute intervals for the entire study period. This experiment was started at 9:30 pm and lasted 11 hours. To provide an autonomic upload function, a fully autonomic data acquisition and uploading system, called “Xenon,” was developed at our laboratory by Prof. Terry B. J. Kuo. The Xenon uploading system consists of two parts, an ultralow power radio frequency (RF) module (Xenon RF module) and its router (Xenon router). The Xenon RF module can be integrated with a BP recorder and can provide an autonomic acquisition and storage function for long-term physiological data via an internet cloud server after relay by the Xenon router.

### Experimental design

For 1 week before participating in the study, the subjects were required to maintain a regular sleep schedule and complete a sleep diary each day. From 3 days before the entire study, the subjects were asked to abstain from caffeine and alcohol and to avoid intense physical activity. During the data collection sessions, all the subjects were randomized in relation to the adaptation day followed by the two experimental conditions: warm (23°C) and cold (16°C). The two experimental conditions were applied more than 1 day apart. The subjects were requested not to consume hot water, soup, drinks, or spicy foods during the study period, and to finish their final meal of the day during the period between 6:00 pm and 7:00 pm. The recording was started at 9:30 pm and lasted 11 hours. The subjects were asked to complete a sleep questionnaire (Pittsburgh Sleep Quality Index) and provide written informed consent prior to participation in the study. During the recording period, the subjects were allowed to undertake an entire night of sleep in the sleep laboratory (Sleep Research Center, National Yang-Ming University, Taiwan); they slept in a sound-attenuated room that was adjusted to either 24.40 ± 0.78°C with 55%–60% humidity for the warm condition or 16.67 ± 0.45°C and 55%–60% humidity for the cold condition. There was no significant difference in humidity between the two conditions. The experimental procedure included baseline recording, an entire night sleep experiment and a cover-to-uncover supine-to-sit test after morning awakening. For the baseline recording, the subjects were allowed to stay awake. They could use a computer, but this excluded playing games, listening music and watching movies. During the entire night sleep experiment, the subjects were requested lie down in a supine position and cover themselves with a quilt. After morning awakening, the subjects underwent the body position changing test, which consisted of the sleep to cover-waking test, the cover to uncover-waking test, and the supine-to-sit test (data not shown). Clothes are an essential aspect of thermal comfort [31]. Thus, the subjects were asked to wear identical clothes during the sleep period and use the same type of bedcover, which we provided to maintain the same level of thermal comfort among all the participants.

### Signal processing

To analyze the HRV, a special computer program in Pascal language (Borland Pascal 7.0, Borland, USA) was designed. The detailed procedures of the computer program are as follows. The ECG signals were preprocessed according to recommended procedures [21], as detailed in our previous investigations [22, 29]. In brief, a computer algorithm identified each QRS complex and rejected each ventricular premature complex or noise according to likelihood based on a standard QRS template. Stationary R-R intervals (RR) were resampled and linearly interpolated at a rate of 64 Hz to provide continuity in the time domain. Acceleration values were stored on the flash drive for each axis, namely X (mediolateral), Y (vertical), and Z (anteroposterior). Each axis had a sampling frequency of 125 Hz and could detect accelerations ranging from -3 to 3 G. A vectorial magnitude for movement was calculated as √X^2^+Y^2^+Z^2^. The magnitude of the physical activities signals (ACT) was quantified by calculating the root mean square (RMS) of the vectorial magnitude for each period (epoch) (data not shown).

### Power spectral analysis

The amplitudes of the HRV were measured in the frequency domain using power spectral analysis. The RR signals to be analyzed were truncated into successive 64-second (4,096 points) time segments (windows or epochs) with a 50% overlap. A Hamming window was applied to each time segment to attenuate the leakage effect [32]. Our algorithm then estimated the power density of the spectral components based on fast Fourier transformation. The resulting power spectrum was then corrected for attenuation resulting from the sampling and the application of the Hamming window [22]. For the HRV analysis, the mean RR, total power (TP), LF (0.04–0.15 Hz), LF to HF (0.15–0.4 Hz) power ratio (LF/HF), normalized LF (LF%), and HF were quantified [21, 22]. The TP, LF, HF, and LF/HF were logarithmically transformed to correct for their skewed distributions [22].

### Sleep scoring analysis

The original data file was converted into European Data Format (.edf) [33] and then imported into a sleep analysis software program (Somnologica 3.1.2, Embla, USA). Computer-assisted sleep analysis was performed according to the criteria defined by Rechtschaffen and Kales [28] and revised by the American Academy of Sleep Medicine. The results were verified by a qualified sleep technician [33]. To clarify the changes of autonomic function during sleep transition, the transition point in this study was defined according to the final epoch of NREM sleep that was followed by the first epoch of REM sleep. The timing of the first epoch of REM sleep that followed the final epoch of NREM sleep before a subject entered REM sleep was used for analyzing sympathetic and parasympathetic activity before and after the sleep transition.

### Statistical analysis

Statistical analyses were performed with SPSS Statistics 20.0 (IBM Corporation, New York, USA), and significance was noted when value of p<0.05. The Shapiro-Wilk test did not show a significant departure from normality in the distribution of investigating values. All data are presented as mean ± standard error mean (mean ± SEM). The time course of variation of HRV indices after the final sleep stage transition from NREM to REM sleep were evaluated by average values, which were estimated by calculating the average absolute values 5 minutes before and after sleep transition. After morning awakening, changes of BP, temperatures, and HRV parameters were estimated by calculating the differential average values during the period 1 hour before and after morning awakening to evaluate the level of MBPS. To address the early-morning BP surge in prehypertensives prior to morning awakening, we calculated the differential average values before (2 to 2.5 hours during an ambient-controlled bedtime period) and after the early-morning surge (5 to 5.5 hours during an ambient-controlled bedtime period). The analysis of the difference between the cold and warm conditions for the normotensives and prehypertensives was performed using a paired *t* test. Pearson's correlation was used to analyze the relationship between MBPS and the differential value of indices of HRV at periods between baseline recording and early sleep and between early sleep and late sleep; p < 0.05 was considered significant.

## Results

### General characteristics of the subjects

In total, 24 young men participated in this study. The demographic data of the prehypertensives showed that the SBP and DBP in the period of awakening during baseline recording were significantly increased compared with normotensives, as was the SBP and DBP in the period of sleep under cold exposure in prehypertensives. The DBP of the baseline awakening of normotensives and the DBP of the bedtime period of prehypertensives were significantly increased under cold exposure compared under the warm condition (Table 1)

### Acute early-morning BP surge and sympathetic changes under cold exposure in prehypertensives

**Fig 1:**
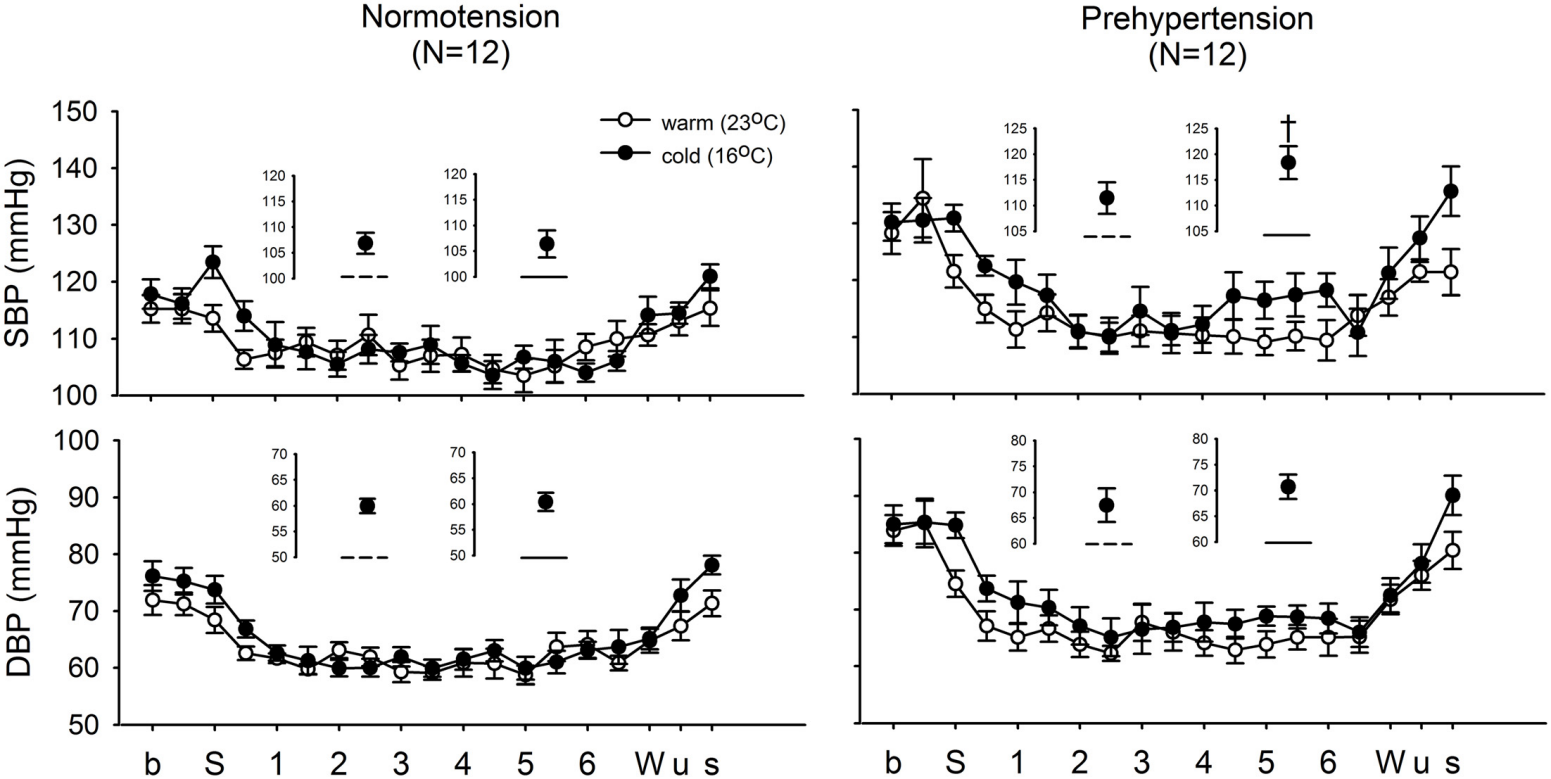
Nighttime pattern of systolic blood pressure (SBP) and diastolic blood pressure (DBP) under cold and warm conditions for normotensives and prehypertensives. From S (sleep, time of lights-off) to W (waking, time of lights-on) is the bedtime period; during this period, we defined the average values from 2 to 2.5 hours and 5 to 5.5 hours as the early and late sleep periods, respectively. The plots of dashed and solid lines in the inserts show the average values for SBP and DBP from 2 to 2.5 hours and 5 to 5.5 hours under warm and cold conditions, respectively. The results show that there is a significant acute early-morning BP surge in SBP from 4 to 6 hours under cold exposure among the prehypertensives (SBP and DBP: 1 recording point/30 minutes). † p < 0.05 for comparison with the average values from 2 to 2.5 hours. Abbreviation: b, baseline recording; S, sleep; W, wake; u, uncover waking; s, sit; numbers 1 to 6 on the X axis indicated the time (hours) relative to S (sleep) state.

**Fig 2:**
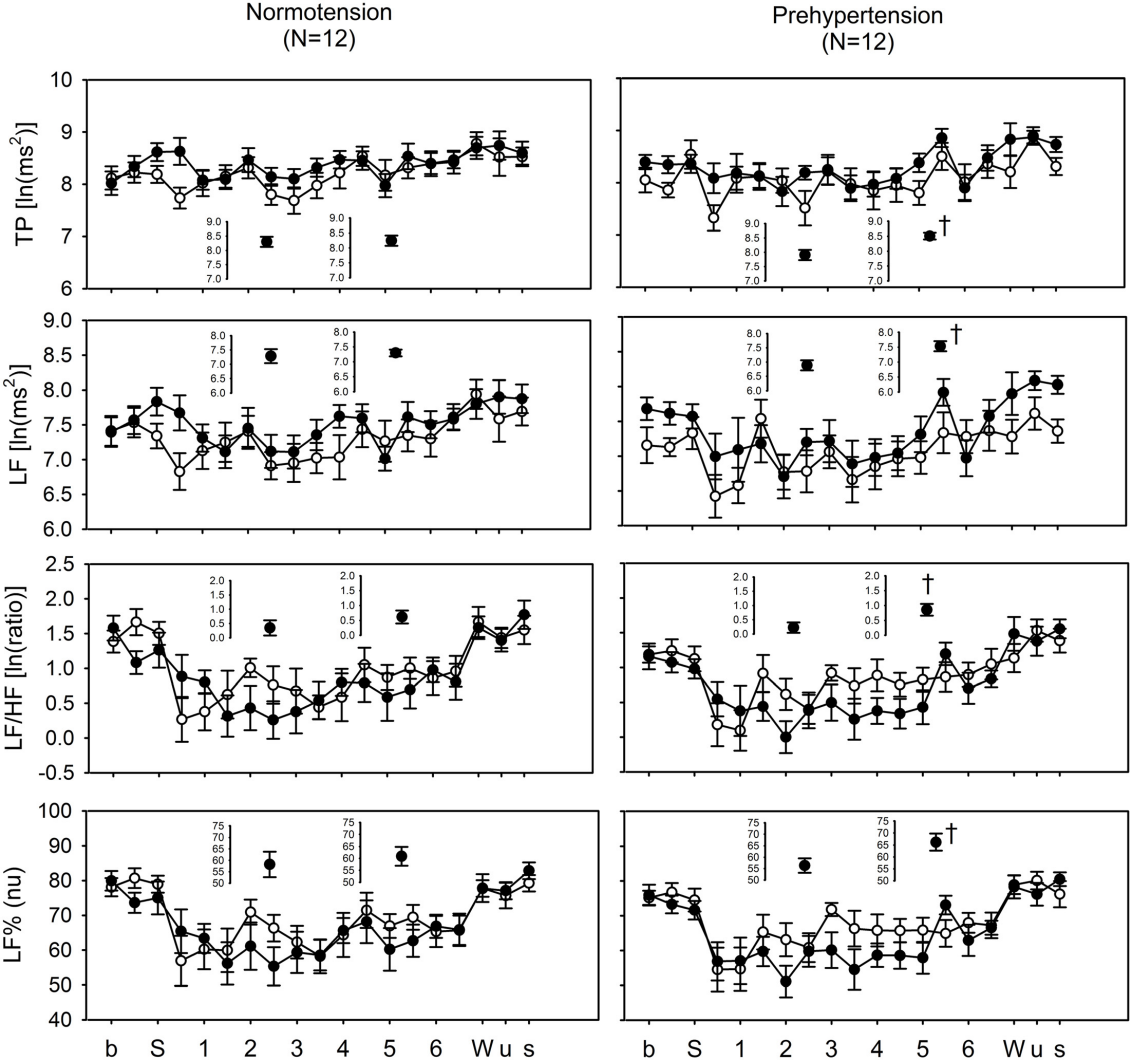
Diurnal patterns of indices of heart rate variability (HRV) for normotensives and prehypertensives under warm and cold conditions over the entire study. Plots of the short dashed and solid lines in the inserts show the average values of HRV indices from 2 to 2.5 hours and 5 to 5.5 hours under warm and cold conditions, respectively. A comparison of the average values of the HRV indices between 2 to 2.5 hours and 5 to 5.5 hours under cold exposure shows that there is a significant early-morning surge in TP, LF, LF/HF, and LF% among prehypertension subjects. † vs. average values from 2 to 2.5 hours, p < 0.05. Abbreviation: b, baseline recording; S, sleep; W, wake; u, uncover waking; s, sit; numbers 1 to 6 on the X axis indicated the time (hours) relative to S (sleep) state; TP, total power of HRV; LF, low-frequency power of HRV; LF/HF, low-frequency power to high-frequency power ratio of HRV; LF%, normalized low-frequency power of HRV.

**Fig 3:**
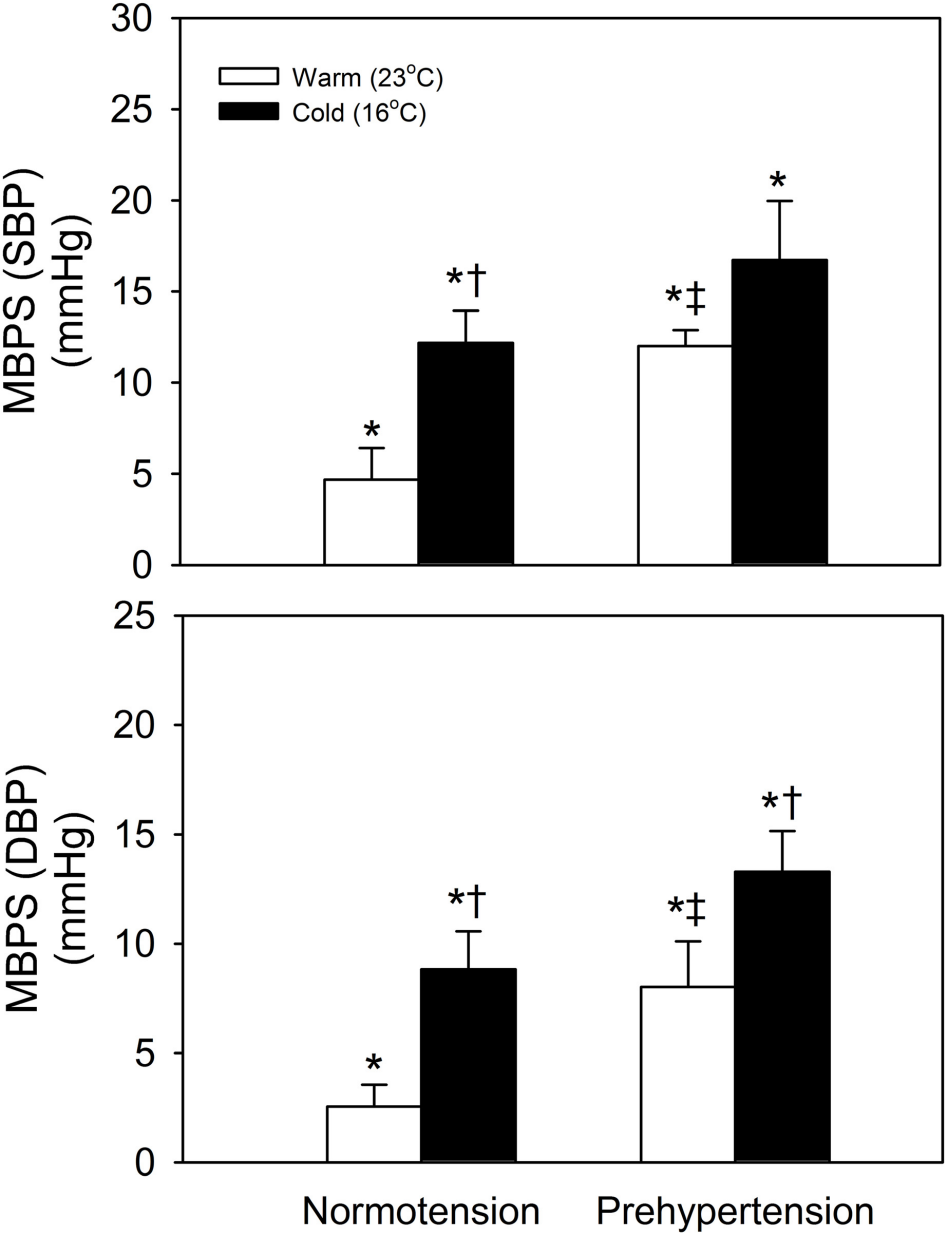
Comparison of the morning blood pressure surge (MBPS) in systolic blood pressure (SBP) and diastolic blood pressure (DBP) under cold and warm conditions between the normotensive and prehypertensive subjects. There are significantly higher MBPS values for SBP and DBP among the normotensive subjects under cold exposure. The prehypertensive subjects exhibited higher MBPS values for SBP and DBP under the warm condition compared with the normotensives. * vs. 0, p < 0.05; † vs. warm condition, p < 0.05; ‡ vs. normotensives, p < 0.05.

The mean ambient temperature under the cold and warm conditions were 16.67 ± 0.45°C and 24.40 ± 0.78°C and the mean difference between the two conditions was 7.73°C (p < 0.001). From S (sleep) to W (wake) is the bedtime period; during this period we defined our average values as being from 2 to 2.5 hours and 5 to 5.5 hours for early and late sleep, respectively (Fig 1). The plots up to the dashed and solid lines on the inserts show the average values of the SBP and DBP from 2 to 2.5 hours and 5 to 5.5 hours during the bedtime period under cold exposure, respectively. Our results showed a significant acute early-morning BP surge from 5 to 5.5 hours under cold exposure for prehypertensives (SBP and DBP: 1 recording point/30 minutes) (Fig 1). We further analyzed autonomic activity during this early-morning surge, revealing a significant early-morning surge in TP, LF, LF/HF, and LF% (Fig 2). Comparing the MBPS after morning awakening under the cold and warm conditions between normotensives and prehypertensives showed a significantly higher MBPS on SBP and on DBP under cold exposure compared with the warm condition for both normotensives and prehypertensives (Fig 3). Our findings also revealed that the MBPS on SBP and DBP under the warm condition for the prehypertensives were significantly higher than for the normotensives (Fig 3). These results indicate that cold exposure exerted a noticeable effect on prehypertensives regarding the early-morning BP surge, which was accompanied by a significant surge in sympathetic activity. In addition, these results also indicate that the MBPS for prehypertensives shows a trend that seems to be higher than that for normotensives, even under the warm condition.

### Significant sympathetic and parasympathetic changes during the final NREM-REM transition under cold exposure in prehypertensives

**Fig 4:**
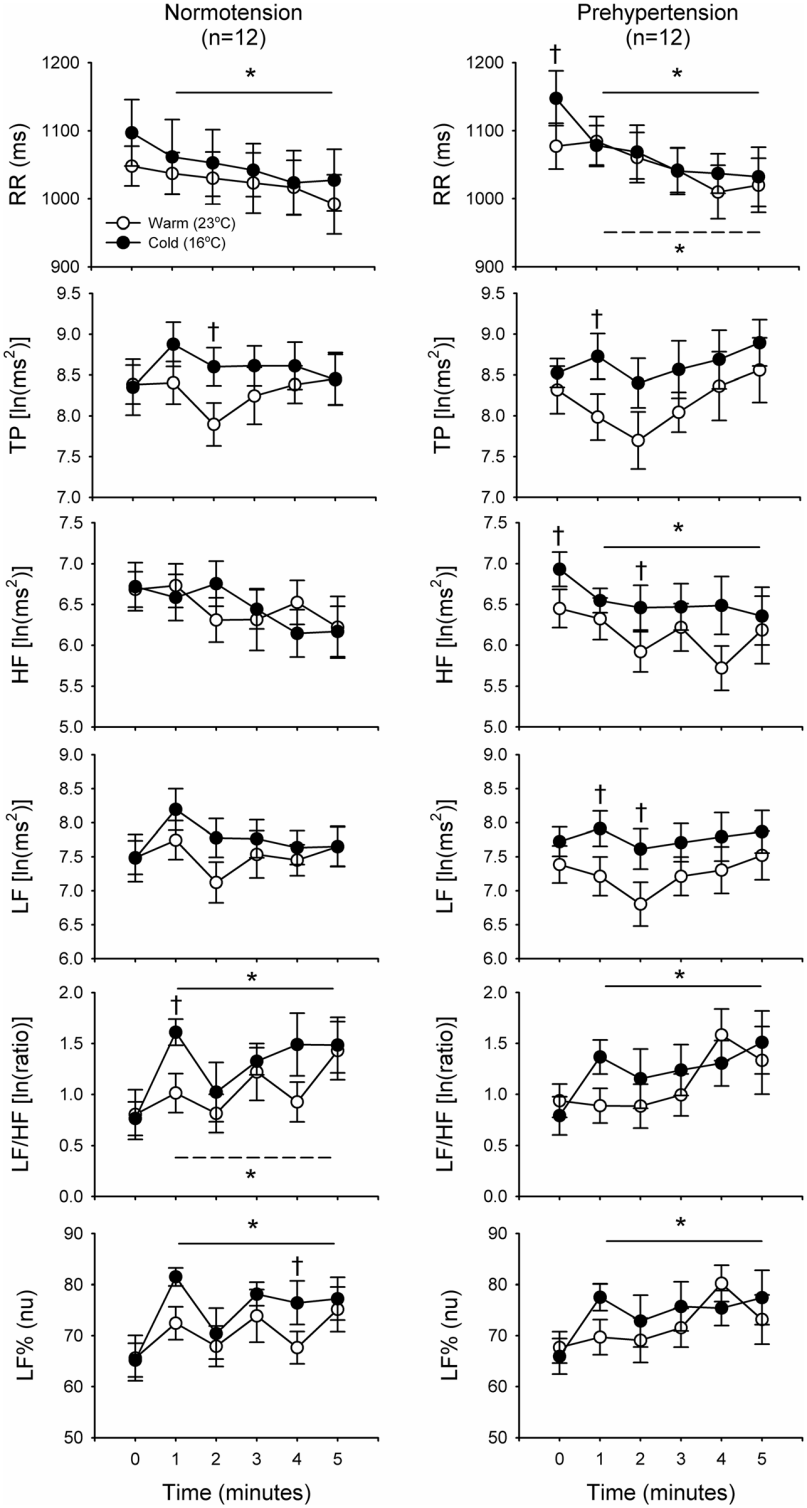
Time course of variation of the heart rate variability (HRV) parameters after the final sleep stage transition from NREM to REM sleep. All dots represent the average absolute values. Dots for point 0 on the X axis represent the average absolute values 5 minutes before the sleep transition, whereas points 1 to 5 represent the average absolute values at 1-minute intervals after the sleep transition. Average values (solid and dashed lines represent the average range) represent the changes after the sleep transition from NREM to REM and were compared with the average values before the sleep transition under the cold and warm condition during REM sleep, respectively. The average values for 5 minutes before transition were compared, both normotensives and prehypertensives under cold exposure after the sleep transition were found to show significant changes in RR, LF%, and LF/HF, with LF/HF and LF% being significantly higher and RR being significantly lower. *p < 0.05 for the average values over 5 minutes after sleep transition compared with the average values over 5 minutes before sleep transition; †p < 0.05 for comparison between the cold and warm conditions. Abbreviation: RR, R-R intervals; TP, total power of HRV; LF, low-frequency power of HRV; LF/HF, low-frequency power to high-frequency power ratio of HRV; LF%, normalized low-frequency power of HRV.

Previous research has reported that there is an exaggerated sympathetic increase during the transition period from NREM to REM sleep under cold exposure [18]. In this study, we further investigated the effect of cold exposure on the transition of sleep stage among prehypertensives and compared this with normotensives. A number of interesting findings emerged from this investigation and these are presented in Fig 4. When the baseline average values for 5 minutes before transition were compared, both normotensives and prehypertensives under cold exposure after the sleep transition were found to show significant changes in RR, LF%, and LF/HF, with LF/HF and LF% being significantly higher and RR being significantly lower (Fig 4). In addition, HF was significantly decreased before the sleep transition in prehypertensives under the cold condition. Compared with the warm condition, there were significantly higher values at some time points for TP, LF/HF, and LF% after the sleep transition in normotensives. Furthermore, some time points for RR and HF before the sleep transition, as well as some time points for TP and LF after the sleep transition, were found to be significantly higher in the prehypertensives (Fig 4).

### Relationship between MBPS and the indices of HRV under the two ambient temperature conditions

**Fig 5:**
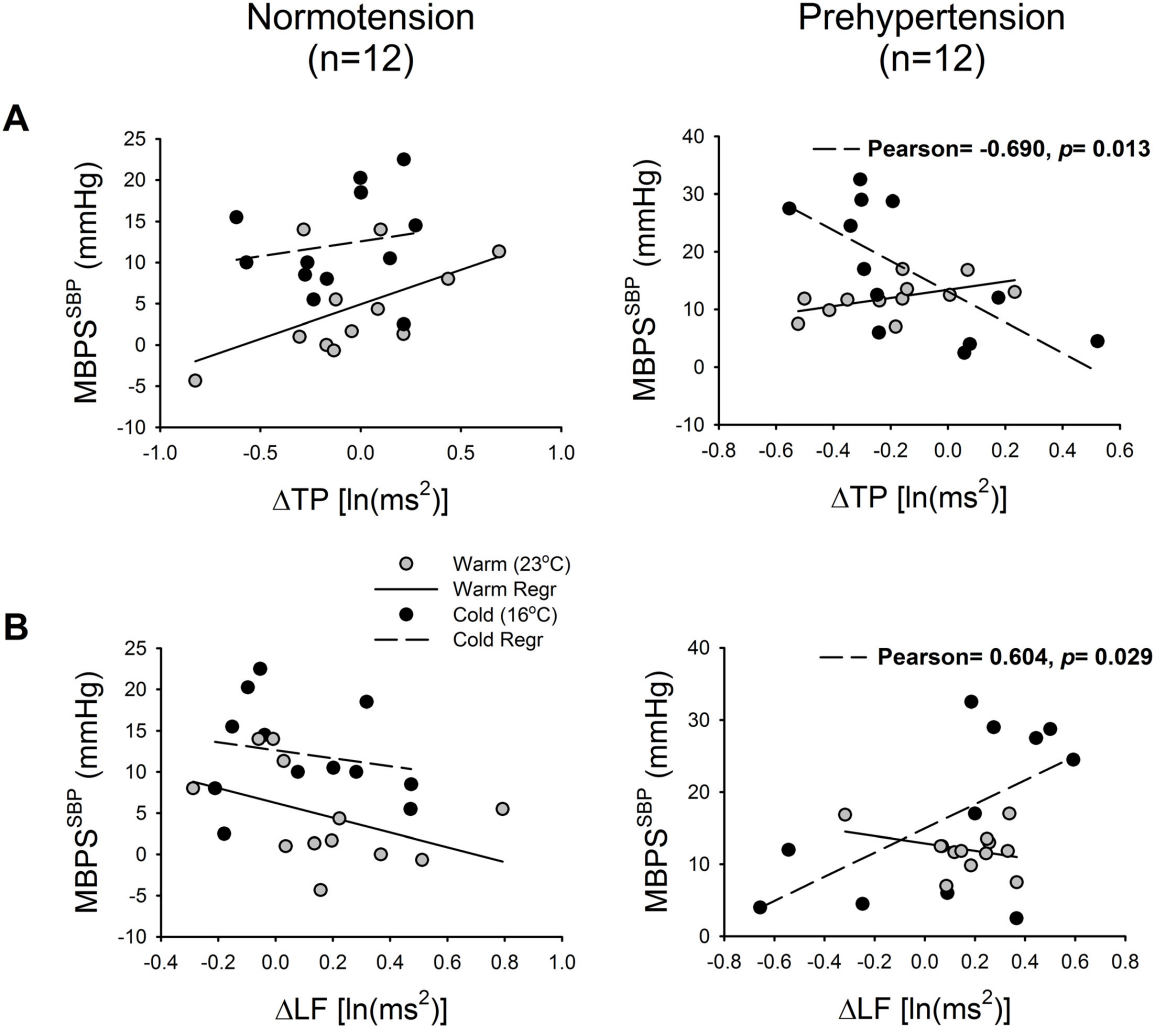
Relationship between the morning blood pressure surge (MBPS) and (A) the differential values of total power of HRV (TP) between early sleep and baseline and (B) low frequency (LF) between late sleep and early sleep. (A) The MBPS and the differential values of TP between early sleep and baseline appear to be highly negatively correlated relative to the change of TP between early sleep and baseline. (B) The relationship between MBPS and the change of LF shows a highly positive correlation: As the change of LF between late sleep and early sleep increases, the MBPS also increases. TP, total power of HRV; LF, low-frequency power of HRV.

We further use correlation analysis to determine the relationship between the magnitude of the MBPS, which is a crucial risk factor associated with stroke, and the changes in the indices of HRV at different sleep stages. The MBPS of the SBP in prehypertensives under cold exposure showed a strong negative correlation with the change between awakening before sleep and early sleep during TP under the cold condition (Pearson's correlation: -0.690, p < 0.05) (Fig 5A), and a positive correlation with the change between early and late sleep for LF (Pearson's correlation: 0.604, p < 0.05) (Fig 5B); however, this was not found when normotensive results were analyzed (Fig 5).

## Discussion

The morning surge of BP is a normal physiological phenomenon during the transition from sleep to morning awakening and it could affect by many factors; these include hypertension, stress [1] and a cold ambient temperature [18]. People with a high normal BP, who are categorized as prehypertensive, have a higher risk of developing hypertension [13] and this is likely related to the development of cardiovascular diseases [14, 15]. Nevertheless, the amplitude of MBPS and the change in autonomic activities during the night sleep period of prehypertensives under cold exposure has not yet been examined. This is the first study to connect HRV, sleep, and MBPS changes among normotensives and prehypertensives. This is also a pioneering study of Taiwanese individuals in the field of changes in autonomic activities during sleep and the MBPS period of normotensives and prehypertensives. During this study, we obtained compelling findings related to prehypertensives and cold exposure, after assessing their autonomic activities and determining their sleep characteristics. Specifically, we found that, before morning awakening, there is an acute early-morning BP surge during the sleep period under cold exposure among prehypertensives and that this is associated with significantly elevations in TP, LF, LF/HF, and LF%. The period of the early-morning surge is during late sleep, which is a more unstable sleep period than early sleep; it includes more REM periods and a greater number of sleep transitions. Therefore, we further analyzed the HRV parameters and the BP changes associated with the transition in sleep stages during late sleep. We found significantly sympathetic changes occur during the final sleep transition from NREM to REM under cold exposure, which is consistent with our previous study [18]. Furthermore, there was also a significant decrease in RR and HF before and after the sleep transition under cold exposure in prehypertensives.

Regarding the early-morning surge, only prehypertensives under cold exposure showed an acute SBP and DBP surge during the early morning (from 4 to 6 hours after lights-off) and before morning awakening. Based on previous studies, the late sleep period before morning awakening is an unstable period during which there are always frequent transitions from sleep to awakening, a higher number of arousals, and longer duration of REM periods [8, 10]. Thus, the late sleep is likely to be a peak risk time for cardiovascular events [8, 9, 34]. The most crucial finding of this study is that there is an acute early-morning surge in SBP under cold exposure 4 to 6 hours after lights-off, and this phenomenon occurs only in prehypertensives. We further analyzed the variation of HRV parameters during this period of the early-morning surge in prehypertensives, revealing a significant early-morning surge in sympathetic activity, including TP, LF, LF/HF, and LF% (Fig 2). Therefore, we believe that the early-morning increase in sympathetic activity and SBP among prehypertensives might be critical factors related to the frequently occurrence of cardiovascular events during this period. The effect of cold exposure on HRV during the entire nighttime sleep was explored by comparing the HRV parameters between normotensives and prehypertensives under cold and warm conditions, showing that under cold exposure among prehypertensives there were significant differences in SBP and DBP during late sleep, which covered the period of the early-morning surge. These findings imply that the early-morning surge during late sleep under cold exposure might be a risk period for cardiovascular events in prehypertensives.

Our results pinpoint the effects of cold exposure on HRV parameters during sleep period. The findings demonstrate that cold exposure may induce an acute elevation in the HRV indices related to sympathetic activity in normotensives and prehypertensives under cold exposure. A surprising finding was that HF, an index of parasympathetic activity, was significantly lower after the sleep transition from NREM to REM sleep, but only among prehyptertensives under cold exposure. Previous findings have indicated that a decrease in parasympathetic cardiac control may cause a disturbance in sympathetic and parasympathetic control among hypertensives [35]. Another previous study indicated that in a mildly cold environment, the hypocretin neuron can modulate changes in BP during both the waking and sleep period [36]. In summary, transitions in sleep stages, which in the present study were from NREM to REM sleep, are associated with changes in cardiac function and changes in BP. These changes seem likely to elevate the risk of cardiovascular events during the NREM-REM transition of late sleep and before morning awakening [1, 37].

Sympathetic activation is associated with hypertension [38]. In prehypertensives, the association between the morning surge in BP and the change in the HRV indices observed for the TP differential values between waking before sleep and after sleep onset are negatively related to the MBPS (Fig 5A); furthermore, the LF between early sleep and late sleep is positively related with the MBPS (Fig 5B). This suggests that the change in HRV might be a predictor of the morning surge in BP. The most likely explanation is that the reduction in TP may be interpreted as being due to impaired regulation of the ANS and its effect on BP during the sleep period. We interpret this to mean that the amplitude of change in the TP between awakening and sleep and in LF between early and late sleep should be able to act as predictors for the morning surge among prehypertensives. Such an explanation may be accounted for, in part, by the autonomic dysfunction affecting BP regulation and the high early-morning surge among prehypertensives.

Potential limitations may have affected this study. First, this study involved only a small population; hence, the findings must be treated with caution. Second, we recruited only young male subjects. Therefore, any generalization of our results to female subjects or to elderly individuals may be limited.

## Conclusions

Our results indicate that the impact of cold exposure may increase the risk of sleep-related cardiovascular events in prehypertensives.
